# Paracentral acute middle maculopathy presenting as a sign of impending central retinal artery occlusion: a case report

**DOI:** 10.1186/s12886-023-02990-6

**Published:** 2023-06-13

**Authors:** Emily Louie, Anthony Tang, Benjamin King

**Affiliations:** Hamilton Eye Institute, University of Tennessee Health Science Center, 930 Madison Avenue, Memphis, TN 38103 USA

**Keywords:** Paracentral acute middle maculopathy, PAMM, Central retinal artery occlusion, CRAO, Deep capillary plexus, Optical coherence tomography, OCT

## Abstract

**Background:**

To report a case of paracentral acute middle maculopathy (PAMM) that progressed to central retinal artery occlusion (CRAO) on spectral domain-optical coherence tomography (SD-OCT).

**Case Presentation:**

A 63-year-old male presented with a paracentral scotoma that began several days ago. His past medical history consisted of third-degree atrioventricular heart block requiring a pacemaker. Giant cell arteritis was unlikely given the patient’s labs, demographics and review of systems. SD-OCT revealed a characteristic hyperreflective band in the inner nuclear layer consistent with PAMM in his left eye. Fluorescein angiography was obtained and was unremarkable. Five days later, the patient developed no light perception in the left eye. SD-OCT showed a diffuse inner retinal hyperreflectivity consistent with CRAO.

**Conclusion:**

PAMM can be a harbinger event for complete CRAO. Complete stroke evaluation should be performed to prevent a cerebrovascular event or progression to complete blindness in the involved eye.

## Background

Central retinal artery occlusion (CRAO) is a thromboembolic event typically resulting in catastrophic and irreversible vision loss [1]. Though some clinical findings – such as Hollenhorst plaques or a history of amaurosis fugax – may suggest higher risk, [2] it is difficult to identify those patients with an impending retinal artery occlusion before it actually occurs. With recent advances in multi-modal imaging, there is an increased interest in using spectral domain optical coherence tomography (SD-OCT) and other modalities to better characterize retinal vascular disease prior to the onset of vision loss.

Paracentral acute middle maculopathy (PAMM) is an SD-OCT finding characterized by hyper-reflective changes within the inner nuclear layer (INL) and inner plexiform layer (IPL) [3]. On infrared reflectance imaging, the corresponding SD-OCT location of lesion can present as a darkened region around the macula [4]. This is thought to arise as a result of microvascular ischemia within the intermediate capillary plexus, which represents a watershed zone due to its more distal location along the retinal vascular pathway [5]. Though not traditionally considered a precursor event to CRAO, PAMM is associated with risk factors that are nearly identical to those of retinal artery occlusion such as age, hypertension and diabetes [6, 7].

In this case, we present a patient who developed acute onset central scotoma with PAMM and subsequently progressed to complete CRAO.

## Case presentation

**Fig. 1:**
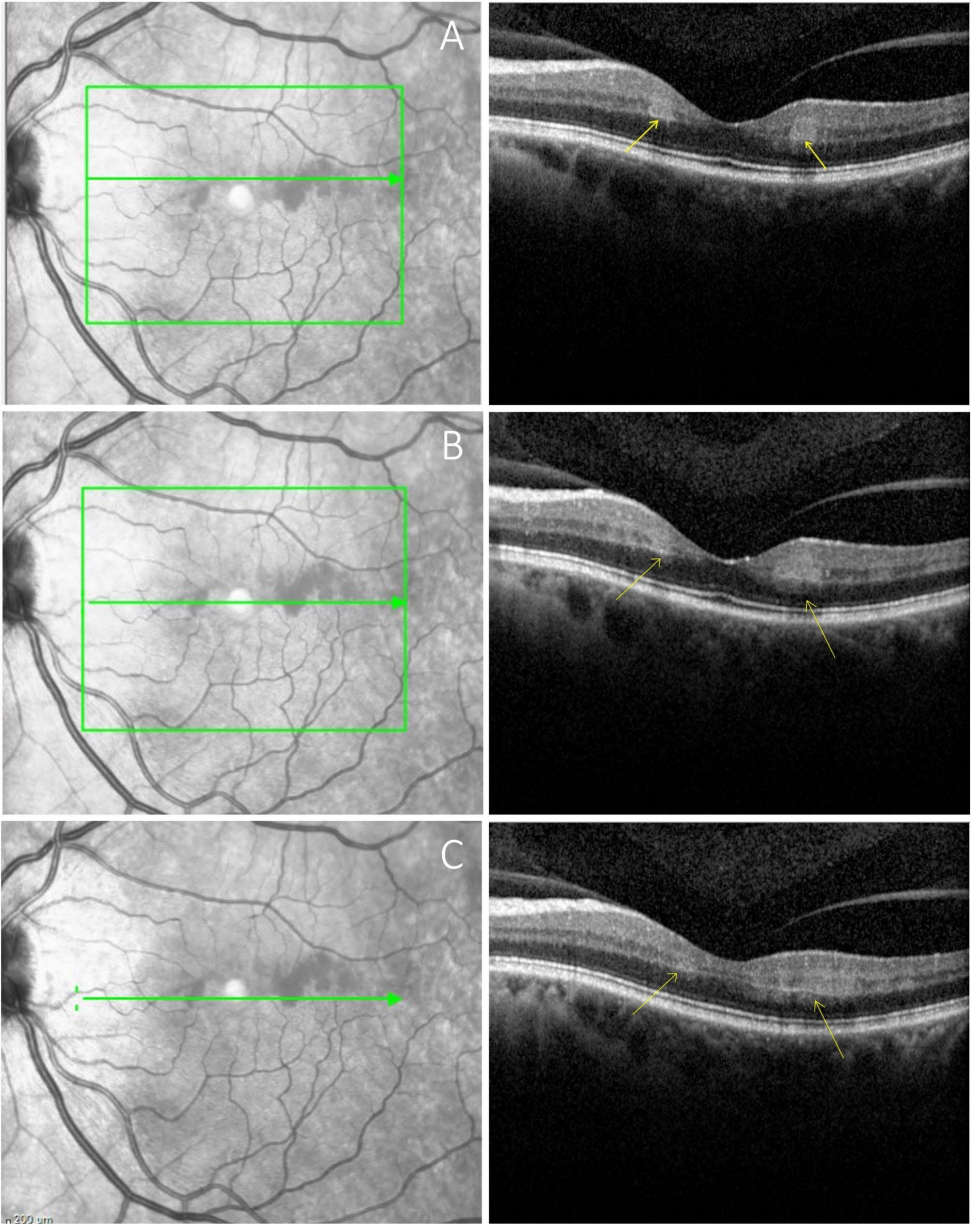
SD-OCT images of the upper (**A**), central (**B**), and lower (**C**) regions of the infarct demonstrating ischemia and hyperreflectivity of the middle and inner retina (yellow arrows) consistent with PAMM. The infrared images (left column) with green arrows indicate the location of the OCT B-scan.

**Fig. 2:**
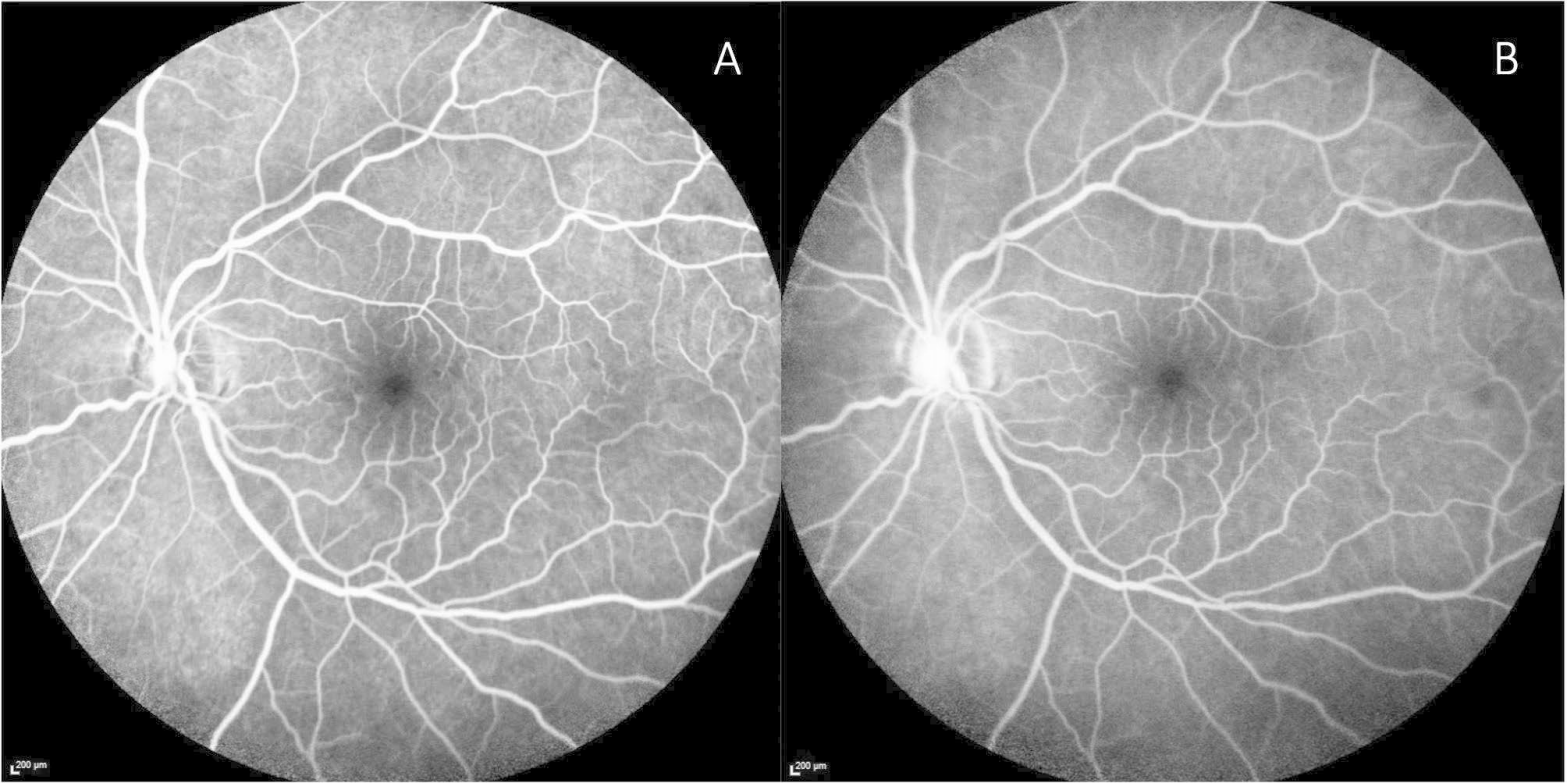
Early (**A**) and late (**B**) phase fluorescein angiography shows normal arterial filling without any signs of retinal vasculopathy

A 63-year-old male presented with a new onset central scotoma in the left eye. Symptoms began approximately 36 h prior when he noted a profound decrease in visual acuity. This had subjectively improved, though at the time of presentation, he could still appreciate what he described as a horizontal line interrupting his central field of vision. He denied any recent headaches, scalp tenderness or jaw claudication. Past medical history was significant for third degree atrioventricular heart block treated with a pacemaker and a single episode of atrial fibrillation for which he was on chronic anticoagulation with apixaban. Visual acuity was 20/25 without correction in both eyes. The fundus exam was unremarkable, though SD-OCT revealed patchy areas of hyper-reflective change involving the INL and IPL which extended temporally from the fovea (Fig. 1). Fluorescein angiography was unremarkable with normal transit time and no areas of arterial attenuation or non-perfusion (Fig. 2). Computed tomography angiography showed mild atherosclerotic changes involving the left carotid artery but no significant stenosis. Erythrocyte sedimentation rate and C reactive protein were both within normal limits.

**Fig. 3:**
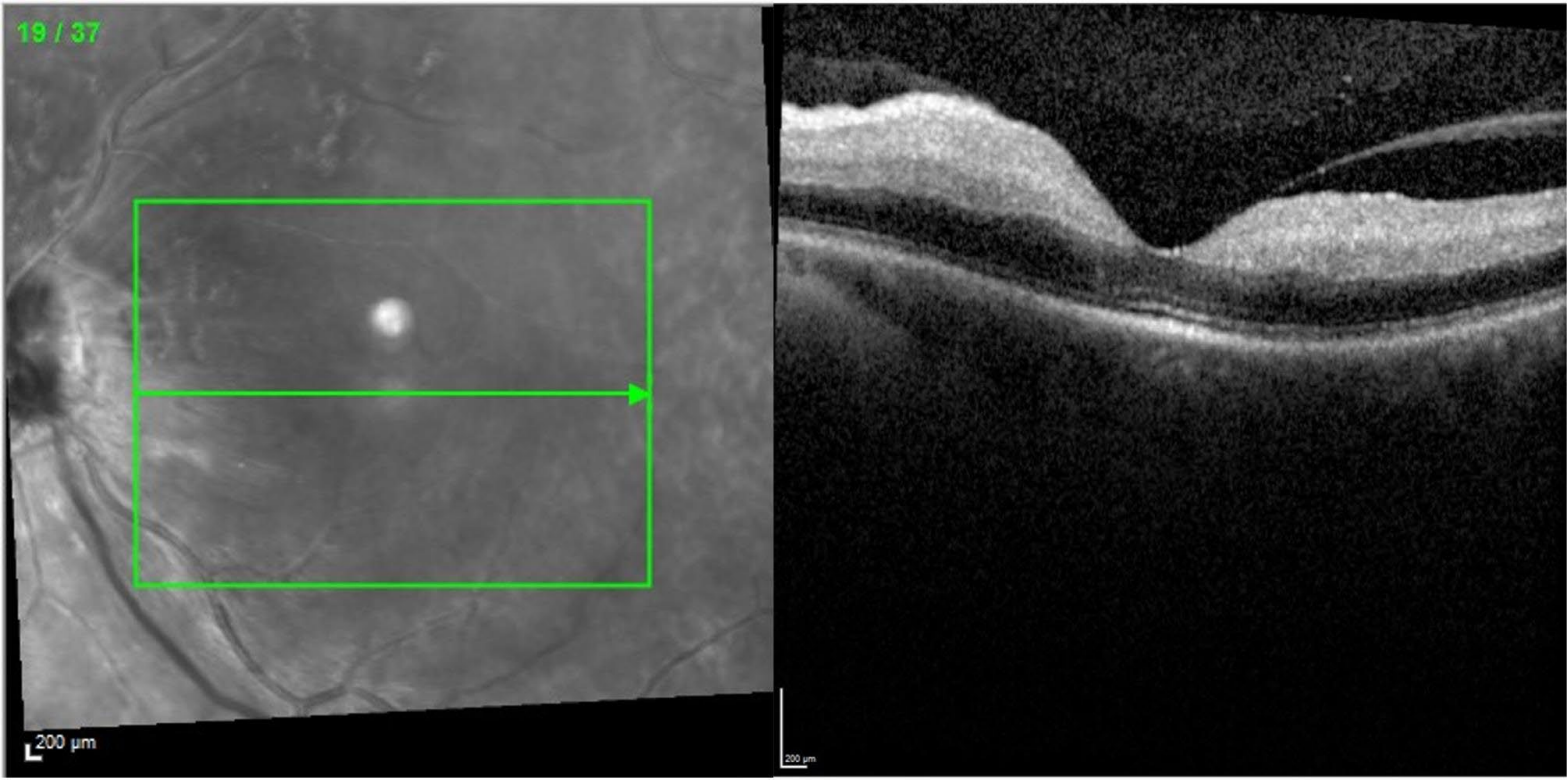
SD-OCT one week later depicting diffuse hyperreflectivity and thickening of the inner retina consistent with complete CRAO. The infrared image with the green arrow indicates the location of the OCT B-scan.

Four days later, the patient noted inferior hemifield vision loss. He was evaluated by neurology but not deemed to be appropriate for thrombolytic therapy with tissue plasminogen activator (tPA). He was prescribed anti-platelet therapy with aspirin 81 mg daily. One day later, he awoke with complete vision loss in the left eye. Visual acuity was hand motion. The fundus exam showed diffuse retinal whitening posteriorly with a cherry red spot though no intravascular plaques or emboli were visible. SD-OCT showed diffuse inner retinal thickening and hyper-reflectivity consistent with complete CRAO (Fig. 3). Carotid ultrasound and trans-esophageal echocardiogram were both unremarkable. Hypercoagulopathy workup was performed in consultation with hematology and was also unremarkable. Warfarin and clopidogrel were initiated while apixaban was discontinued. After the event, the patient was lost to follow-up.

## Discussion and conclusions

Here we present a case of PAMM occurring five days before complete CRAO. There have been multiple reports of PAMM identified in the setting of incomplete CRAO [8, 9]. Our case is unique in that none of the clinical findings of retinal artery occlusion – retinal whitening, arterial attenuation or delayed filling on angiography – were present on the initial exam. The PAMM lesion described here likely resulted from a separate thromboembolic event which spontaneously resolved so that the area of retinal infarction was limited to that most susceptible to watershed ischemia. This is in contrast to middle maculopathy in the setting of incomplete CRAO where ischemic lesions appear more diffusely throughout the macula in either a globular or perivenular pattern [10].

PAMM has been reported in association with multiple different causes including sickle cell disease, hypercoagulable state, medication use and intraocular surgery [11]. PAMM has also been reported in systemic diseases such as idiopathic intracranial hypertension, meningitis and giant cell arteritis [4, 12, 13]. Arterial hypoperfusion likely represents the common pathogenic etiology whereby microvascular ischemia within the most distal areas of the retinal arterial system results in localized infarction of the INL and adjacent plexiform layers [10]. This watershed infarct hypothesis is especially applicable in our case where the PAMM lesion occurred specifically along the horizontal raphe which represents the border zone separating the superior and inferior retinal vascular territories. We suspect therefore that the findings in this case represent a spontaneously resolving thromboembolic event resulting in a localized watershed infarct and this was subsequently followed by a separate event culminating in complete arterial occlusion.

It is well established that PAMM occurring in the setting of incomplete CRAO may progress to complete CRAO [4, 10, 14]. Significant risk factors foreshadowing this catastrophic event were not evident. It is possible that the embolic source originated from isolated cardiac emboli secondary to atrial fibrillation. An unremarkable echocardiogram may not necessarily rule out smaller emboli which may still cause complete CRAO. Our case demonstrates that even a smaller, more localized PAMM lesion without accompanying signs of arterial hypoperfusion may precede the development of complete CRAO. PAMM has been reported in otherwise healthy patients without risk factors for CRAO; this prompts the question of whether any therapeutic intervention may be indicated at initial presentation which might prevent this outcome [4]. Zhao et al. report a case where PAMM lesions resolved after starting anti-platelet therapy with aspirin. Our patient also began taking aspirin after PAMM was diagnosed, but this did not prevent onset of CRAO [14]. Intra-arterial tPA has shown some efficacy in reversing vision loss if administered within 12 h of onset [15]. However, this has not yet been validated in a clinical trial and it is difficult to quantify when the risk of impending CRAO is high enough to justify thrombolytic therapy in this setting.

In summary, we present a case of PAMM immediately preceding complete CRAO. Future investigation is needed to better establish the risk correlation between these two events. For now, it is clear that PAMM may represent a precursor event heralding the onset of catastrophic vision loss. As such, it reinforces the critical importance of an urgent diagnostic workup to identify a thromboembolic source and, in the event of a negative workup, to consider anti-platelet or even thrombolytic therapy if suspicion is high.

## Data Availability

All data generated or analyzed during this study are included in this published article.

## Abbreviations

PAMM: Paracentral acute middle maculopathy
CRAO: Central retinal artery occlusion
SD-OCT: Spectral domain optical coherence tomography
INL: Inner nuclear layer
IPL: Inner plexiform layer
tPA: Tissue plasminogen activator
